# Pyruvate Induces Transient Tumor Hypoxia by Enhancing Mitochondrial Oxygen Consumption and Potentiates the Anti-Tumor Effect of a Hypoxia-Activated Prodrug TH-302

**DOI:** 10.1371/journal.pone.0107995

**Published:** 2014-09-25

**Authors:** Yoichi Takakusagi, Shingo Matsumoto, Keita Saito, Masayuki Matsuo, Shun Kishimoto, Jonathan W. Wojtkowiak, William DeGraff, Aparna H. Kesarwala, Rajani Choudhuri, Nallathamby Devasahayam, Sankaran Subramanian, Jeeva P. Munasinghe, Robert J. Gillies, James B. Mitchell, Charles P. Hart, Murali C. Krishna

**Affiliations:** 1 Radiation Biology Branch, Center for Cancer Research, National Cancer Institute, Bethesda, Maryland, United States of America; 2 H. Lee Moffitt Cancer Center and Research Institute, Tampa, Florida, United States of America; 3 Radiation Oncology Branch, Center for Cancer Research, National Cancer Institute, Bethesda, Maryland, United States of America; 4 National Institute of Neurological Diseases and Stroke, Bethesda, Maryland, United States of America; 5 Threshold Pharmaceuticals, South San Francisco, California, United States of America; University of Oxford, United Kingdom

## Abstract

**Background:**

TH-302 is a hypoxia-activated prodrug (HAP) of bromo isophosphoramide mustard that is selectively activated within hypoxic regions in solid tumors. Our recent study showed that intravenously administered bolus pyruvate can transiently induce hypoxia in tumors. We investigated the mechanism underlying the induction of transient hypoxia and the combination use of pyruvate to potentiate the anti-tumor effect of TH-302.

**Methodology/Results:**

The hypoxia-dependent cytotoxicity of TH-302 was evaluated by a viability assay in murine SCCVII and human HT29 cells. Modulation in cellular oxygen consumption and *in* *vivo* tumor oxygenation by the pyruvate treatment was monitored by extracellular flux analysis and electron paramagnetic resonance (EPR) oxygen imaging, respectively. The enhancement of the anti-tumor effect of TH-302 by pyruvate treatment was evaluated by monitoring the growth suppression of the tumor xenografts inoculated subcutaneously in mice. TH-302 preferentially inhibited the growth of both SCCVII and HT29 cells under hypoxic conditions (0.1% O_2_), with minimal effect under aerobic conditions (21% O_2_). Basal oxygen consumption rates increased after the pyruvate treatment in SCCVII cells in a concentration-dependent manner, suggesting that pyruvate enhances the mitochondrial respiration to consume excess cellular oxygen. *In vivo* EPR oxygen imaging showed that the intravenous administration of pyruvate globally induced the transient hypoxia 30 min after the injection in SCCVII and HT29 tumors at the size of 500–1500 mm^3^. Pretreatment of SCCVII tumor bearing mice with pyruvate 30 min prior to TH-302 administration, initiated with small tumors (∼550 mm^3^), significantly delayed tumor growth.

**Conclusions/Significance:**

Our *in* *vitro* and *in* *vivo* studies showed that pyruvate induces transient hypoxia by enhancing mitochondrial oxygen consumption in tumor cells. TH-302 therapy can be potentiated by pyruvate pretreatment if started at the appropriate tumor size and oxygen concentration.

## Introduction

Regions of low oxygen concentration (hypoxia) within solid tumors arise due to the structural and functional abnormalities of tumor vasculature [1]. The chaotic nature of tumor vascular networks leads to two kinds of hypoxia: 1) diffusion-limited or chronic hypoxia, and 2) cycling or acute hypoxia [2]–[5]. It is well recognized that hypoxia is implicated in resistance to radiotherapy and to conventional chemotherapy, both of which are more effective in normoxic regions [6]–[8]. A significant proportion of radiation-induced DNA damage requires oxygen, making radiotherapy less effective in hypoxic regions [9]–[10]. Poor perfusion in hypoxic regions can also limit drug penetration and confer resistance to chemotherapy [11]. While the presence of both chronic and acute hypoxia in tumors can limit successful treatment by radiotherapy and/or chemotherapy [12]–[13], such conditions in turn provide a basis for the use of hypoxia-activated cytotoxins [14].

Research on bioreductive prodrugs, a class of agents that can be selectively activated within a hypoxic tumor microenvironment, has generated a series of anti-tumor compounds generally classified as hypoxia-activated prodrugs (HAPs) since their original description in 1980 [15]. TH-302 consists of a 2-nitroimidazole trigger covalently linked to bromo isophosphoramide mustard (Br-IPM) effector moiety, which is currently in Phase III trials for the treatment of soft tissue sarcoma in combination with doxorubicin, and of pancreatic cancer with gemcitabine, as well as in earlier stage trials in both solid tumors and hematologic malignancies such as leukemia and multiple myeloma [16]–[19]. One-electron reduction of TH-302 by bioreductive enzymes can generate the corresponding free radical anion, which is restored to its original state under aerobic conditions *via* reoxidation by molecular oxygen, leading to superoxide production *via* futile redox cycling. TH-302 exhibits minimal toxicity in aerobic normal tissue. In contrast, hypoxic conditions facilitate fragmentation of the prodrug to release the Br-IPM moiety (**Figure S1 in File S1**) [20]. Once activated, the Br-IPM moiety can efficiently induce intramolecular 1′ 3′-cross-linkage of DNA, resulting in S139 phosphorylation of histone H2AX and ultimately causing tumor cell death [20]–[21].

Our lab has developed imaging capabilities to serially map tumor oxygen *in* *vivo* and changes in tumor pO_2_ distribution in response to treatment using electron paramagnetic resonance imaging (EPRI) [22]–[24]. Studies from our lab and others using such imaging techniques have found that tumors display both spatial and temporal heterogeneities in pO_2_ status. Additional studies have shown that these tumors can be rendered significantly more hypoxic for a period of up to 5 hours by a bolus administration of pyruvate [25]; administration of pyruvate prior to radiotherapy in an SCCVII xenograft model was found to impose radioresistance. These findings suggest that the combined use of pyruvate and TH-302 may enhance the therapeutic efficacy of TH-302 in solid tumors.

In this study, the hypoxia-dependent activation of TH-302 was evaluated in murine squamous cell carcinoma SCCVII cells and human colon adenocarcinoma HT29 cells under the experimentally created hypoxic conditions. The changes in tumor oxygenation elicited by pyruvate treatment were validated by real-time monitoring of cellular oxygen consumption *in* *vitro* and EPR oxygen imaging *in* *vivo*. The potential enhancement of anti-tumor effect of TH-302 in combination with pyruvate was assessed by the growth suppression of tumor xenografts in mice, with further characterizations of the treatment response carried out by immunohistochemical analysis, immunoblotting, and MRI measurements.

## Materials and Methods

### Ethics Statement

All animal experiments were carried out in compliance with *the Guide for the Care and Use of Laboratory Animals* (National Research Council, 1996) and approved by the National Cancer Institute Animal Care and Use Committee (NCI-CCR-ACUC (Bethesda), Protocol# RBB-159) [26].

### Cell culture

The murine squamous cell carcinoma SCCVII cell line was obtained from Dr. T. Philips, University of California San Francisco (UCSF). The SCCVII is a squamous carcinoma which arose spontaneously in the abdominal wall of a C3H mouse in the laboratory of Dr. H. Suit, Massachusetts General Hospital (Boston, MA) [27]–[28], and was subsequently adapted for clonogenic growth by Dr. K. Fu, UCSF [29]. The human colon cancer cell line HT29 was purchased from American Type Culture Collection (ATCC). Both cells were tested in 2013 by IDEXX RADIL (Columbia, MO) using a panel of microsatellite markers (Table S3 in File S1). All cell lines were maintained in RPMI 1640 supplemented with 10% fetal calf serum and antibiotics. The cells were maintained in a humidified chamber at 37°C containing 5% CO_2_.

### Cell viability assay

The viability of cells exposed to TH-302 (Threshold Pharmaceuticals, USA) under varied percentages of oxygen was assessed using the colorimetric 3-(4,5-dimethylthiazo-2-yl)-2,5-diphenyl-tetrazolium bromide (MTT) assay. SCCVII or HT29 cells (5×10^3^/100 µl/well) were plated into a 96-well microplate and incubated at 37°C/5% CO_2_ overnight. Various concentrations of drugs (100 µL) were added and incubated under aerobic (21% O_2_) or hypoxic (0.1% O_2_) conditions in a sealed chamber at 37°C/5% CO_2_ for 2 h. HT29 cells were incubated for an additional 24 h at 37°C/5% CO_2_. Cells were washed and cultured for 12 h (SCCVII) or 48 h (HT29). To measure cell viability, 10 µl of MTT reagent (5 mg/mL) was added and the cells were incubated for 2–3 h to form formazan crystals. After removing the medium, crystals were dissolved in 100 µL DMSO and the cell viability was determined by measuring optical density at 560 nm. The drug concentration resulting in 50% growth inhibition (IC_50_) was calculated using a nonlinear regression curve fitting (sigmoidal dose-response) in Prism 4 (GraphPad Software Inc., La Jolla, CA).

### Measurement of oxygen consumption rates (OCR)

The basal oxygen consumption rates [OCR (pmol/mg protein)] in SCCVII and HT29 cells were measured using the Seahorse XF96 Extracellular Flux Analyzer (Seahorse Bioscience, Billerica, MA). Five thousand (SCCVII) or forty thousand (HT29) cells were plated into each well of a 96-well plate and cultured overnight. Cells were treated with sodium pyruvate (0.2, 0.5 and 2 mM) and OCR changes were monitored. To confirm that pyruvate was entering the mitochondria and stimulating the electron transport chain (ETC), cells were treated with 1 µM of rotenone and antimycin A, inhibitors of complex I and III in the ETC, respectively, before and after 2 mM pyruvate treatment.

### Animal studies

Female 5–8 week old C3H/Hen mice (20–34 g) and athymic NCr-nu/nu nude mice (strain 01B74) (19–25 g) were supplied by the Frederick Cancer Research Center, Animal Production Department (Frederick, MD). Formation of mouse SCCVII and human HT29 solid tumors and the management of mice during imaging were carried out as previously described [25], [30]. Isotonic pyruvate solution (pH 7.4, 1.15 mmol/kg), prepared by dissolving 30 µL of pyruvic acid in 4.5 ml of alkaline solution (40 mM HEPES, 94 mM NaOH, 30 mM NaCl, 100 mg/L EDTA), was intravenously injected into mice tail vein [25], and 100 mg TH-302 (Threshold Pharmaceuticals) per kg body weight (100 mg/kg) dissolved in Dulbecco’s Phosphate-buffered Saline (DPBS) was intraperitoneally injected 30 min after pyruvate injection. The pyruvate/TH-302 combination was injected on three consecutive days following tumor implantation (days 7/8/9 or 9/10/11 for SCCVII and days 8/10/12 or 14/16/18 for HT29).

### EPR and MR imaging with pyruvate and TH-302 injection

Technical details of the EPR scanner operating at 300 MHz, data acquisition based on a single-point imaging modality, image reconstruction, and the oxygen mapping procedure were described in an earlier report [22]. The EPRI measurements were carried out before and 30 min after isotonic pyruvate (pH 7.4, 1.15 mmol/kg) injection into mice with SCCVII or HT29 xenografts at different tumor sizes (500–1500 mm^3^). At 30 min after pyruvate injection, mice were intraperitoneally injected with 100 mg/kg of TH-302. MRI experiments were conducted using a 7T scanner controlled with ParaVision 5.1 (Bruker BioSpin MRI GmbH, Ettlingen, Germany) and a 17 mm diameter parallel coil resonator in which only the tumor-bearing leg is inserted. After a quick assessment of the sample position by a fast low-angle shot (FLASH) tripilot sequence, T_2_-weighted anatomical images were obtained using a fast spin echo sequence (RARE) with: matrix = 256×256, echo time (TE) = 13 ms; repetition time (TR) = 2500 ms; RARE factor = 8. T_2_ maps were generated from images obtained by a multi-slice multi-echo (MSME) sequence with: matrix = 128×256; 10 echo train of 10 ms TE steps; TR = 2000 ms. Diffusion-weighted spin echo images were obtained with: matrix = 256×256; TE = 22.5 ms; TR = 2000 ms. Apparent diffusion coefficient (ADC) was separately acquired with 3 different diffusion gradient directions (read, phase, and slice), and the average was used as ADC value. The parametric images including T_2_ and ADC maps were generated using a code written in MATLAB (Mathworks, Natick, MA) or ImageJ (http://rsb.info.nih.gov/ij/) using the MRI analysis calculator plug-in (Karl Schmidt, HypX Laboratory, Brigham and Women’s Hospital, Boston, MA).

### Immunohistochemical analysis

Tumor tissues were excised 24 or 1 h after intravenous injection of pimonidazole or a FAM-FLIVO™ in vivo apoptosis reagent (Immunochemistry Technologies, LLC, Bloomington, MN), respectively, in accordance with the manufacturer’s instructions. The tumors were frozen by ultra-cold ethanol, sectioned to 10-µm thickness using a cryostat, and sections were thaw-mounted on glass slides. After fixing with 4% paraformaldehyde, sections were treated with cold acetone for 30 min. After blocking nonspecific binding sites on sections with Protein Block Serum-Free reagent (Dako North America Inc., Carpinteria, CA) for 30 min, the slides were submerged in Rb PhosphoDetect™ Anti-H2AX (pSer^139^) antibody (Calbiochem/Millipore, Billerica, MA; 1∶800) overnight at 4°C. The sections were incubated with an Alexa Fluor 546 F(ab’)_2_ fragment of goat anti-rabbit IgG (H+L) (Invitrogen/Life Sciences, Grand Island, NY; 1∶2000) and Hypoxyprobe 4.3.11.3 mouse MAb (hpi; 1∶250) for pimonidazole staining for 2 h at room temperature, and then mounted with Prolong Gold antifade reagent with DAPI (Invitrogen). Fluorescence microscopy was performed using an Axiovert 200 inverted Fluorescent microscope (Carl Zeiss, Thornwood, NY), and images were captured using Image-Pro Plus Version 4.0 imaging software (Media Cybernetics, Rockville, MD). To quantify the pimonidazole or H2AX (pSer^139^) stained areas, digital images of stained sections at 4.2× magnification were assembled to compose a whole tumor image, and stained pixels were counted using ImageJ. Percentages of γH2AX are calculated from the intensity of the positive area of each image.

### Immunoprecipitation and immunoblotting

SCCVII tumor-bearing C3H/Hen mice were intravenously injected with isotonic pyruvate (pH 7.4, 1.15 mmol/kg), and 30 min later, intraperitoneally injected with TH-302 (100 mg/kg). One day later, tumors were excised, fragmented and digested with a solution of 2 mg/ml collagenase (Sigma-Aldrich) and 0.2 mg/ml DNase (Sigma-Aldrich, St. Louis, MD). After cell counting, cell samples were rinsed with PBS, suspended with hypotonic buffer in a Nuclear Extract Kit (Active Motif, Carlsbad, CA) and incubated for 15 min on ice. Samples were treated with detergent, vortexed and centrifuged at 13,000×g for 30 s. The pellet was further treated with reagents according to the manufacturer’s instructions for γH2AX detection. The Ser139 phosphorylated γH2AX was detected using HRP-conjugated pSer139 γH2AX antibody and the band intensity was calculated (n = 3). The supernatant was subjected to immunoprecipitation using protein G-linked dynabeads (Invitrogen) and rabbit anti-cleaved caspase-3 antibody (Cell Signaling Technology, Danvers, MA) according to the manufacturer’s instructions.

All samples were treated with SDS sample buffer and incubated at 95°C for 3 min. Protein samples of equal amounts were separated via SDS-PAGE on 18% Tris-glycine acrylamide gels (Novex-Invitrogen). Following transfer to nitrocellulose, samples were probed with primary antibodies (1∶1000), followed by the appropriate HRP-conjugated secondary antibodies diluted to 1∶2000, and visualized by chemiluminescence (Perkin-Elmer, Akron, OH). To confirm equal protein loading and transfer, membranes were stripped with ReBlot Plus (Chemicon/Millipore) and reprobed using anti-actin antibody (or other control protein antibody). Densitometric analysis was accomplished with Image Analyzer software coupled with the Fluorchem FC800 system (Alpha Innotech, San Leandro, CA). Density values for each protein band were normalized to the volume of each tumor.

### Statistical analysis

All results were expressed as the mean ± SE. The differences in means of groups were determined by the 2-tailed Student’s *t* test.

## Results

### Cell viability assay

**Figure 1:**
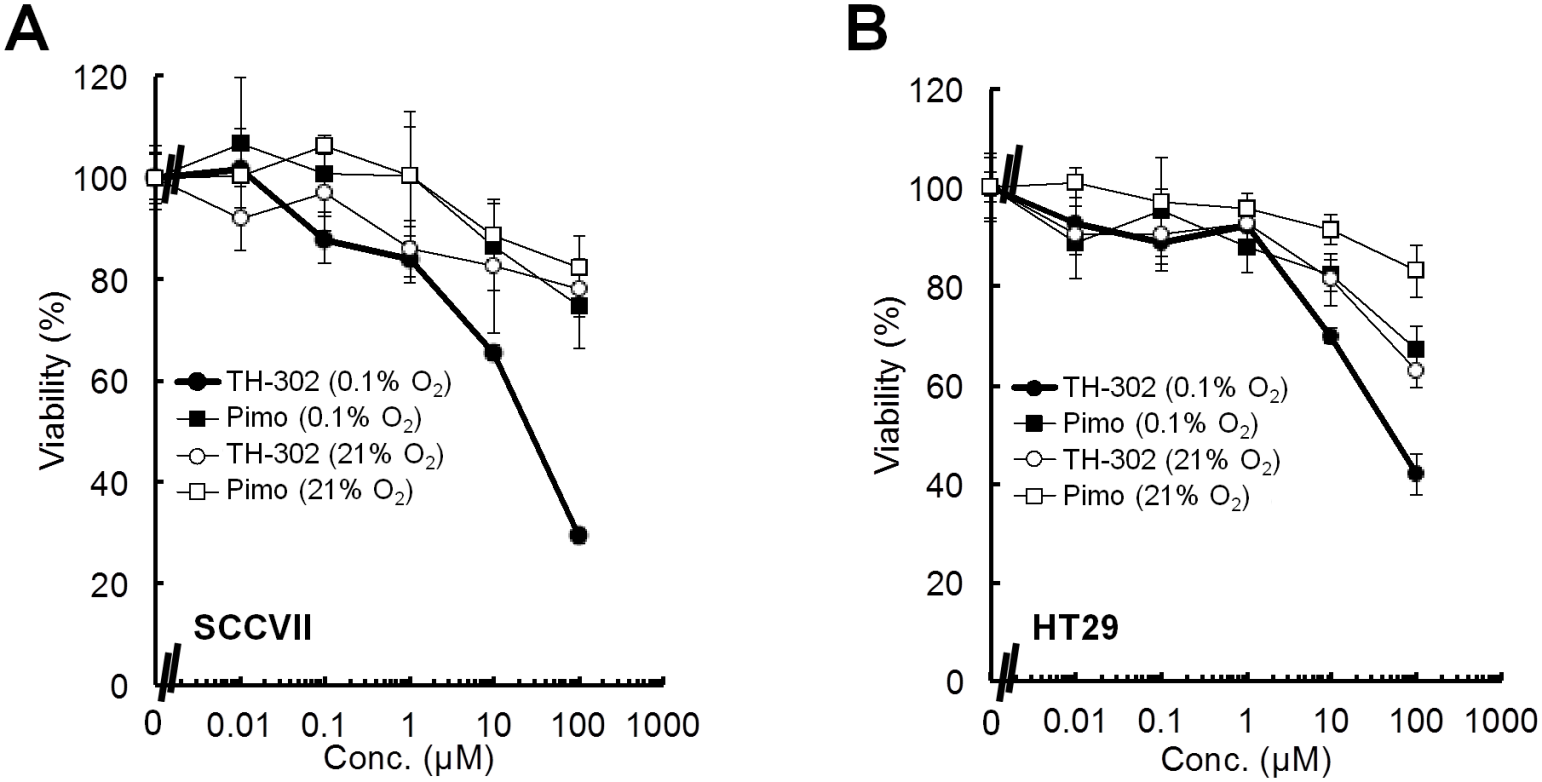
Cell proliferation following a 2 h treatment of SCCVII and HT29 cells with varying concentrations of TH-302 under aerobic (21% oxygen) or hypoxic (0.1% oxygen) conditions. **A, B,** Cell proliferation of SCCVII (**A**) or HT29 (**B**) cells following a 2 h treatment with varying concentrations of TH-302 or pimonidazole (pimo) at different levels of oxygen. Data are from 3 experiments; error bars represent the SE.

To evaluate the hypoxia-dependent activation of TH-302, the viability of SCCVII and HT29 cells following incubations with various concentrations of TH-302 under aerobic (21%) and hypoxic (0.1%) conditions was measured using the MTT assay. The growth of both SCCVII and HT29 cells was minimally inhibited under aerobic conditions, similar to cells treated with pimonidazole, a widely used hypoxia marker for immunohistology which has an oxygen sensitive 2-nitroimidazole part but not a cytotoxic Br-IPM effector part (**Figure 1**, **Figure S1 in File S1**). By contrast, TH-302 strongly inhibited the growth of both SCCVII and HT29 cells under hypoxia. The IC_50_ under hypoxia [IC_50_ (0.1% O_2_)] was 31.2±8.1 µM for SCCVII and 79.4±28.6 µM for HT29 cells, respectively. The IC_50_ under aerobic conditions [IC_50_ (21% O_2_)] was estimated to be >200 µM for both cell lines. These results suggest that TH-302 is significantly activated under hypoxic microenvironment to release the Br-IPM effecter moiety, thereby inhibiting cell growth.

### Influence of pyruvate on cellular oxygen consumption

**Figure 2:**
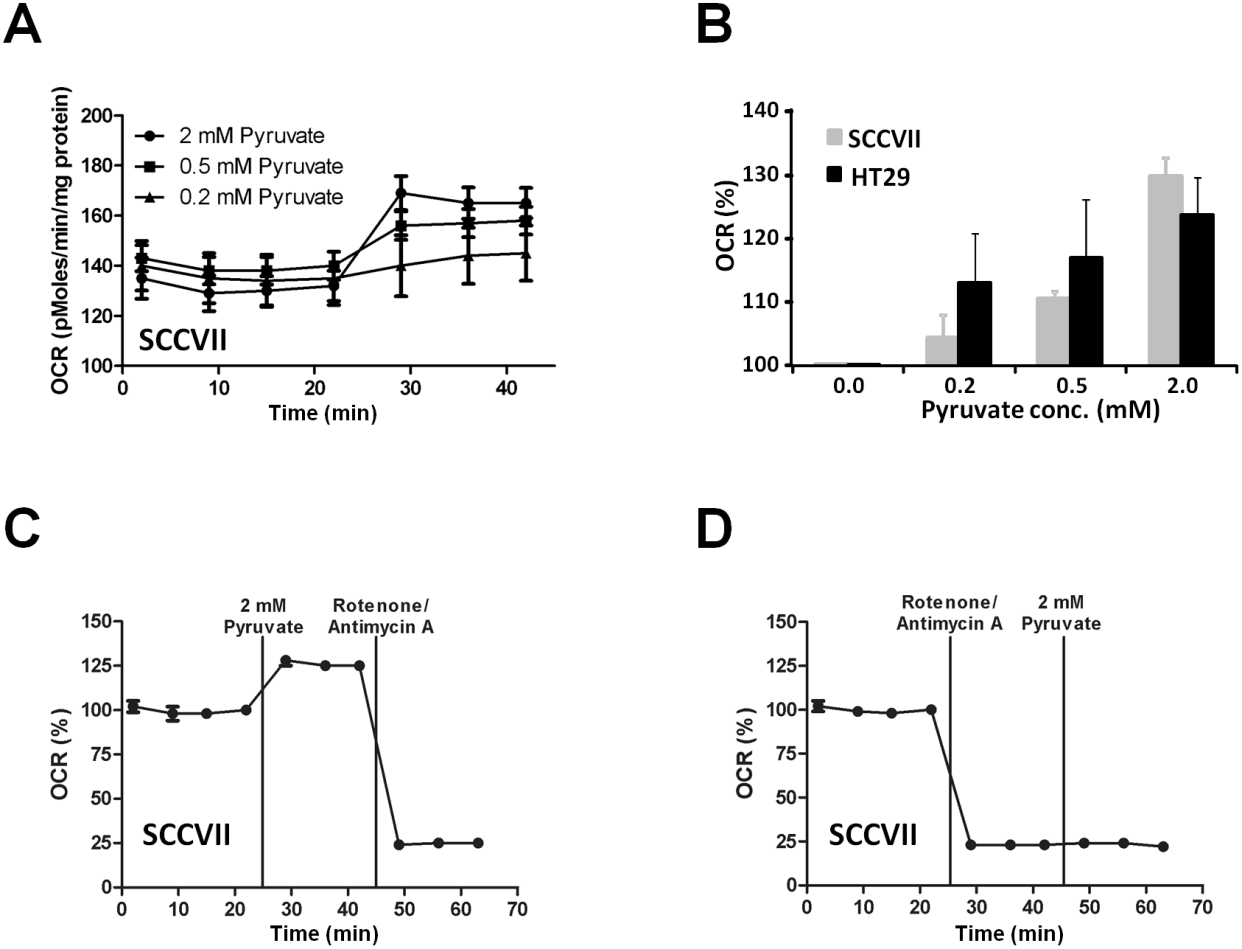
Monitoring the oxygen consumption rate (OCR) in SCCVII and HT29 cells using XF96 Analyzer (Seahorse Bioscience). **A, C**, OCR trace before and after the treatment with 0.2, 0.5 or 2 mM of exogenous pyruvate. **B,** OCR increase ratio (%) after the treatment of SCCVII and HT29 cells with each concentration of pyruvate. **C**, D, OCR response by the treatment of SCCVII with 2 mM pyruvate before (C) and after (D) incubating the inhibitors (antimycin A and rotenone) of mitochondrial respiratory chain.

The Seahorse XF96 Extracellular Flux Analyzer measures real-time oxygen consumption rates [OCR (pmol/mg protein)] and was used to monitor the mitochondrial respiration of SCCVII and HT29 cells. The basal OCR of SCCVII cells was 137–142 pmol/mg protein (**Figure 2A**), which increased following administration of pyruvate (0.2, 0.5 and 2 mM) in a concentration-dependent manner (3–26% increase) (**Figure 2B**). Likewise, the basal OCR increased after pyruvate treatment in HT29 cells and the pyruvate-induced change of the OCR was similar to SCCVII cells. To confirm that pyruvate was entering the mitochondria and stimulating the electron transport chain (ETC), SCCVII cells were treated with 2 mM pyruvate followed by 1 µM Rotenone and Antimycin A, which inhibit complexes I and III in the ETC, respectively. The OCR was significantly decreased by both rotenone and antimycin A (**Figure 2C**). Similarly, the OCR of SCCVII cells pre-treated with rotenone and antimycin A did not increase in response to 2 mM pyruvate (**Figure 2D**). Pyruvate did not show any effect on the MTT viability assay in either SCCVII or HT29 cells (**Figure S2 in File S1**). These results suggest that pyruvate enhances mitochondrial respiration and enforces consuming more cellular oxygen.

### Influence of pyruvate on tumor oxygen level

**Figure 3:**
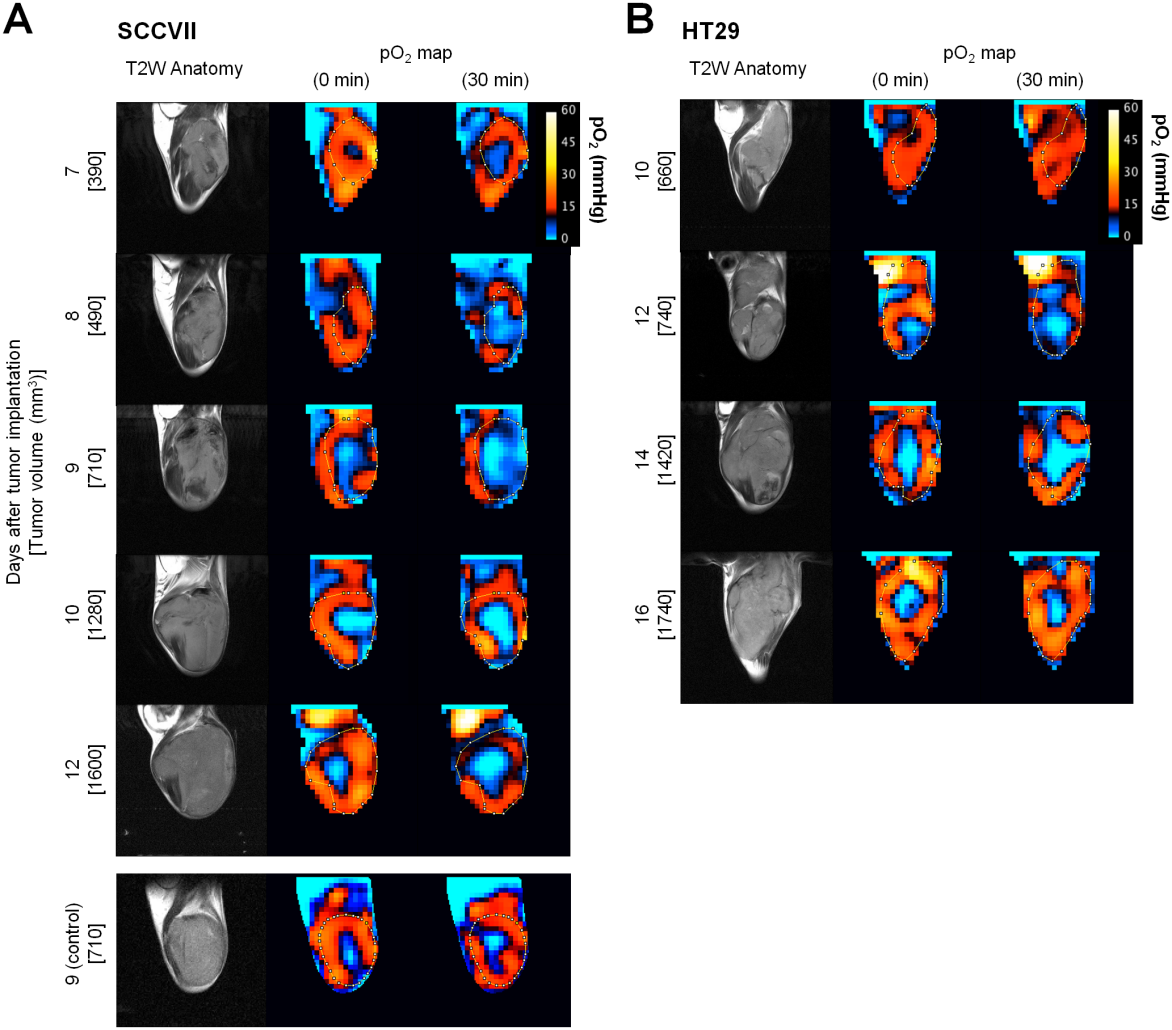
Noninvasive monitoring and quantification of tumor median pO_2_ by EPRI and MRI before and after pyruvate injection in SCCVII and HT29 tumors. **A, B**, T_2_-weighted anatomical image (*left*) and pO_2_ maps measured before (*middle*) and 30 min after (*right*) pyruvate (or vehicle for control group) injection into SCCVII (**A**) or HT29 (**B**) tumor-bearing mice at 7–12 and 10–16 days, respectively, after tumor implantation.

To examine the extent of hypoxia existing in the SCCVII and HT29 xenografts and the effect of pyruvate injection on the tumor oxygen level *in* *vivo*, EPRI experiments were conducted at different tumor sizes (500–1500 mm^3^). The tumor anatomical images were obtained by T_2_ weighted MRI, followed by EPR oxygen imaging before and after injection of pyruvate. **Figure 3A** shows the results from SCCVII tumors in C3H mice at different tumor sizes. The left column in **Figure 3A** shows anatomic images of the maximal center slice of tumor-bearing leg (**Figure S3 in File S1**) acquired on days 7, 8, 9, 10 and 12 after tumor implantation, showing an increase in tumor size as a function of time after implantation. The images in the middle and right columns are pO_2_ maps on the same days, prior to and 30 min after a bolus administration of pyruvate (1.15 mmol/kg). The pO_2_ images (middle column) show that the pO_2_ distribution in these tumors is spatially heterogeneous with regard to regions of hypoxia (pO_2_<10 mmHg) and normoxia. When pO_2_ scans were obtained 30 min after pyruvate administration, a global decrease in tumor pO_2_ was observed in tumors of all sizes (right column), consistent with earlier data showing that pyruvate enhances cellular oxygen consumption *in* *vitro* (**Figure 2**). The administration of vehicle alone did not influence the pO_2_ levels (Figure 3A). Similar results were obtained from MRI and EPRI experiments using HT29 xenografts (**Figure 3B**).

**Figure 4:**
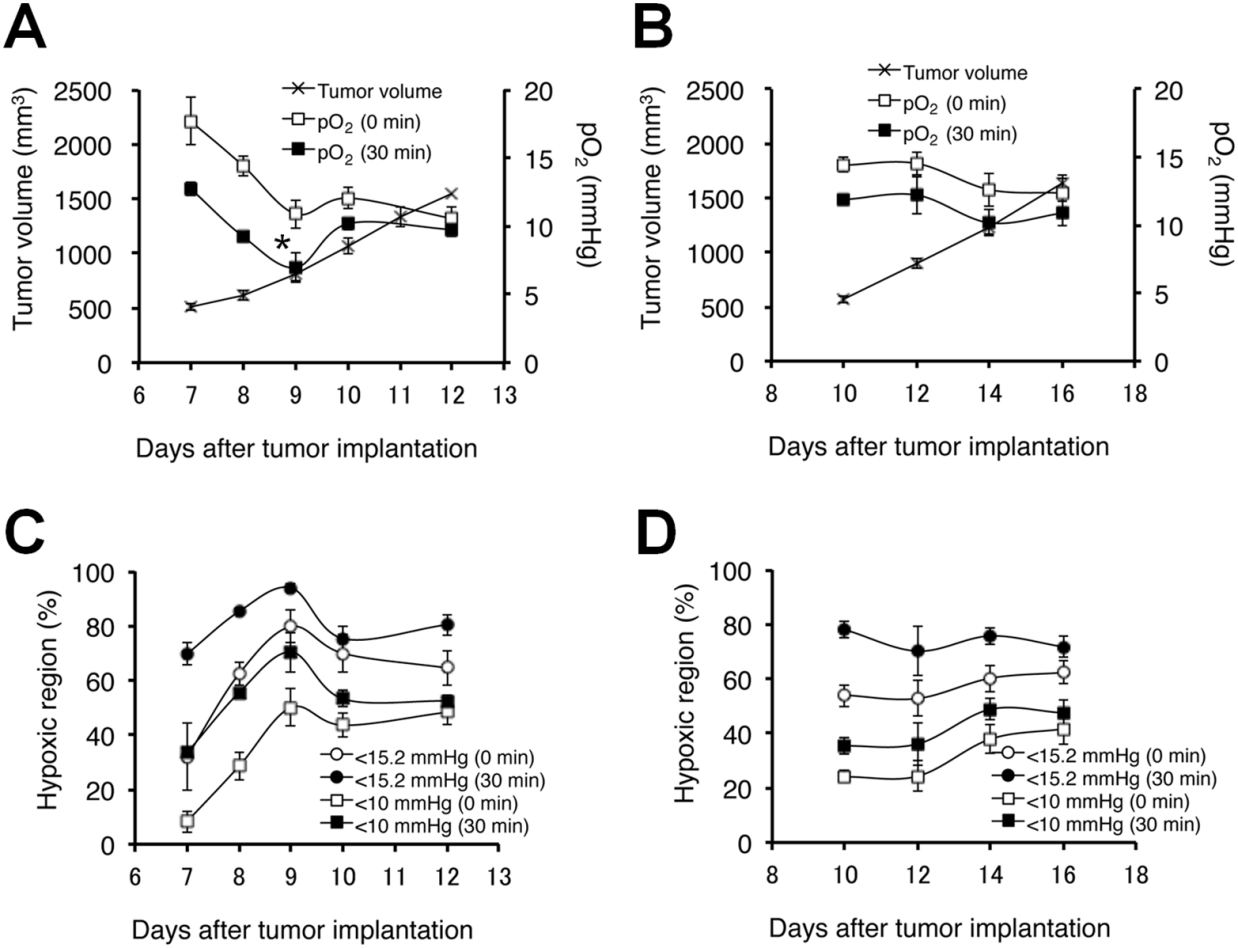
Quantitation of median pO_2_ changes and proportional hypoxic regions. **A, B**, Median pO_2_ profile before (0 min) and 30 min after pyruvate injection in tumors of 500–1500-mm^3^ size on SCCVII (**A**) and HT29 (**B**) tumors. **C, D**, Proportional hypoxic fraction (HF) at pO_2_<15.2 or <10 mmHg on SCCVII (**C**) and HT29 (**D**) tumors. Each value was measured across three 2-mm slices from the center of the 3D image. The values shown are the mean ± SE. *, *P*<0.05 as compared with the pO_2_ (30 min after pyruvate administration) on day 10. The number of animals at each time point is provided in Table S1 in File S1.

The results obtained from SCCVII (**Figure 3A**) and HT29 (**Figure 3B**) tumor-bearing mice were analyzed quantitatively and the results presented in **Figures 4A–D** **, Tables S1** and **S2 in File S1**. **Figure 4A** shows the tumor volume (mm^3^) on days 7 to 12 after implantation of SCCVII tumor. The tumor volume increased from 500 to 1500 mm^3^ during this time window. At baseline, the median pO_2_ in the tumors decreased from 17.7±1.8 to 10.6±0.8 mmHg with increasing tumor size (**Table S1 in File S1**). By comparison, the pO_2_ decreased after pyruvate administration in tumors of all sizes, with larger differences in smaller tumors (Δ3.7–7.3 mmHg in ∼620 mm^3^ tumors). Similar trends, though of a smaller magnitude, were recorded for HT29 tumor-bearing mice, where a size dependent decrease in median pO_2_ (14.5±0.5 to 12.4±1.1 mmHg) was observed and hypoxia was accentuated by pyruvate administration (Δ0–4.6 mmHg in 500–1500 mm^3^ size) (**Figure 4B**).

To examine the global changes in the extent of hypoxia caused by bolus pyruvate administration, the percentage of hypoxic fraction (HF) at 15.2 mmHg and 10 mmHg before and after pyruvate administration was determined in the tumor xenografts (**Figures 4C and 4D** **, Tables S1** and **S2 in File S1**). Approximately 8% of the pixels showed a pO_2_<10 mmHg, which increased to approximately 33% after pyruvate administration in the SCCVII tumors at day 7, while the tumor fraction displaying pO_2_<15.2 mmHg was 32% before pyruvate administration and 70% after pyruvate administration (**Figure 4C** **, Table S2 in File S1**). At day 12, the hypoxic fraction at the 10 mmHg level did not significantly change after pyruvate administration (**Table S2 in File S1**). However, the hypoxic fraction at the 15 mmHg level on day 12 increased by pyruvate administration, although to a lesser degree than on day 7 (**Table S2 in File S1**). HT29 tumors showed a similar size dependent increase in hypoxia (**Figure 4D** **, Table S2 in File S1**), and pyruvate administration resulted in an increase in the hypoxic fraction after 30 min. As with the SCCVII tumors, the magnitude of increase in hypoxia was greater in smaller tumors, and this was true at pO_2_ levels of both 10 and 15 mmHg. Overall, serial imaging studies of SCCVII and HT29 xenografts show that SCCVII tumors displayed a greater change in hypoxia when challenged with a pyruvate bolus.

### Effect of pyruvate-induced transient tumor hypoxia for TH-302 treatment

**Figure 5:**
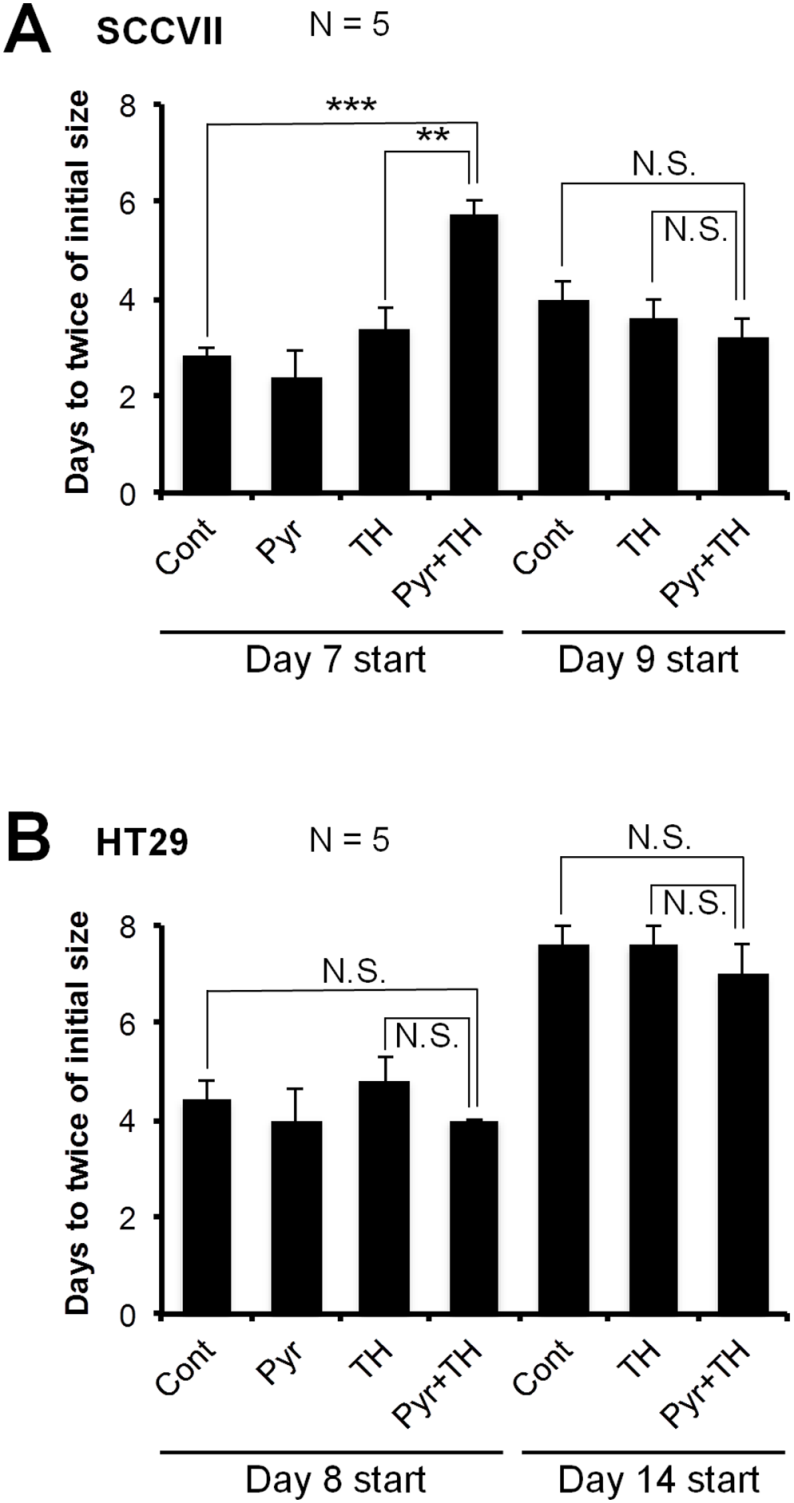
Tumor doubling times after three treatments with pyruvate and/or TH-302. Doubling time was measured as the increase from initial tumor size, which was the tumor volume prior to treatment. **A**, SCCVII tumor-bearing C3H/Hen mice. Mice were treated three times on days 7–9 (Day 7 start) or days 9–11 (Day 9 start). **B**, HT29 tumor-bearing athymic nude mice. Mice were treated three times on days 8/10/12 (Day 8 start) or days 14/16/18 (Day 14 start). Data are mean ± SE of 5 mice. **, *P*<0.01, ***, *P*<0.001; N.S., not significant.

Based on the results from the imaging data where pyruvate induced hypoxia in SCCVII tumors and, to a lesser extent in HT29 tumors, the therapeutic efficacy of pyruvate/TH-302 combination therapy was evaluated using SCCVII-bearing C3H mice and HT29-bearing athymic NCr-nu/nu nude mice. **Figure 5A** shows the doubling time of SCCVII tumors in mice treated three times with pyruvate or TH-302 alone, or TH-302 30 min after pyruvate injection on days 7/8/9 (Day 7 start), or days 9/10/11 (Day 9 start). As compared with the untreated control or TH-302 alone groups, administration of TH-302 30 min after pyruvate injection significantly delayed the SCCVII tumor growth by the treatment on days 7–9 when pyruvate infusion induces larger magnitude of decrease in tumor pO_2_ (<10 mmHg on day 8 and 9) and increase in hypoxic fraction (>50% on days 8 and 9) (**Figure 4A, C** **, Table S1, S2 in File S1**). Pyruvate itself showed no observable side effects (**Figure S4 in File S1**). Tumor growth was not inhibited when the treatment started on days 9–11, where the extent of tumor pO_2_ response to pyruvate pretreatment was smaller than when the treatment initiated at early stage of tumor on days 7–9. These data suggest that smaller volume (∼550 mm^3^) with lesser pO_2_ (<10 mmHg) and larger hypoxic fraction (>50%) are required to significantly delay SCCVII tumor growth by TH-302 treatment. On the other hand, neither treating HT29 tumors with pyruvate/TH-302 on days 8/10/12 (Day 8 start) nor days 14/16/18 (Day 14 start) significantly extended the doubling time as compared with control or TH-302 monotherapy (**Figure 5B**). This is consistent with the fact that HT29 tumors grow more slowly and have higher pO_2_ than SCCVII tumors, even after pyruvate infusion (**Figure 4B** **, Table S1 in File S1**). Taken together, these data suggest that the combination of pyruvate/TH-302 was effective within the time period when pO_2_ levels are decreased to ≤10 mmHg by pyruvate injection (**Figure 4A, B**).

**Figure 6:**
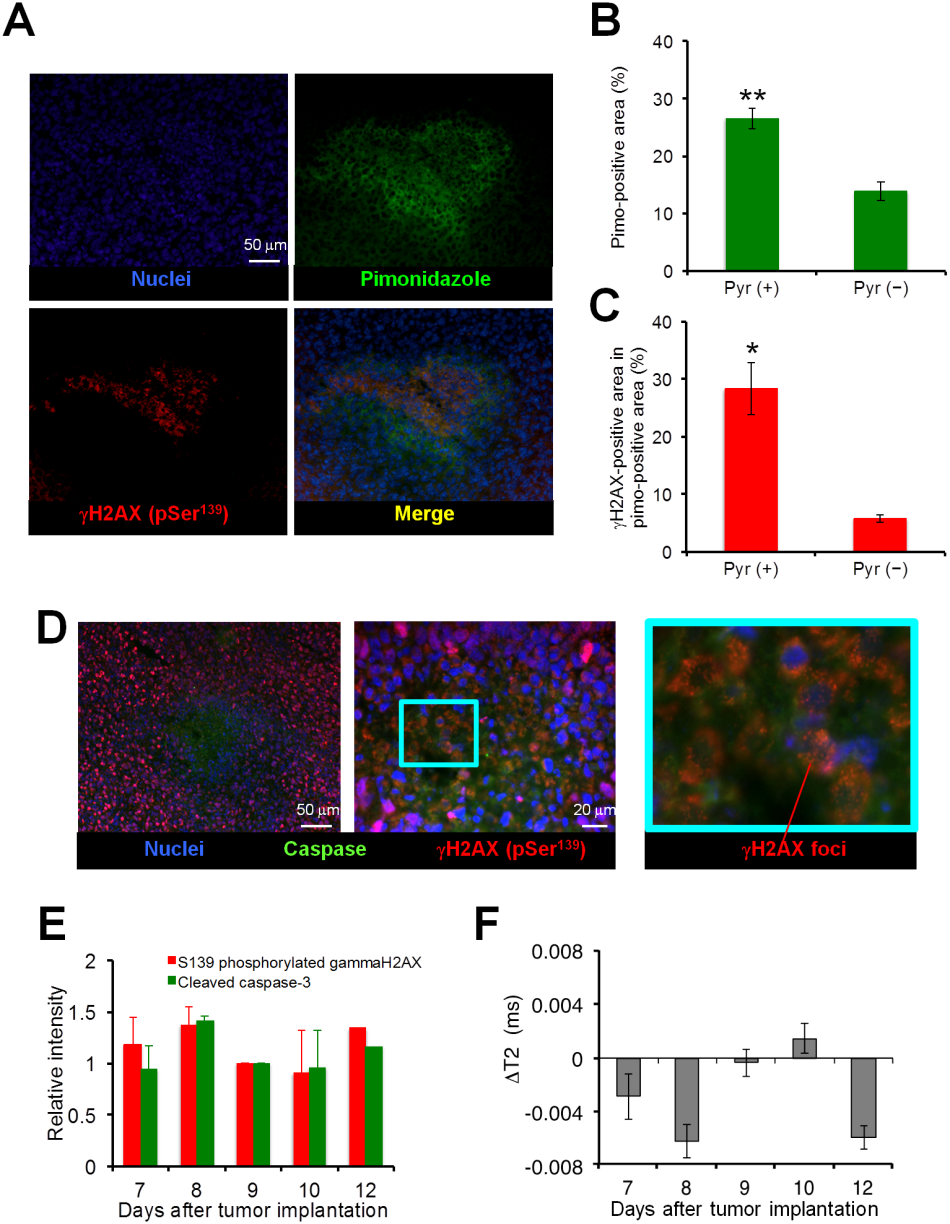
**A**, Representative images of hypoxic areas in tumors detected by pimonidazole binding (green) and the phosphorylation of S139 (pSer^139^) in histone H2AX (red). A bolus dose of pyruvate (1.15 mmol/kg) was intravenously injected, and pimonidazole (60 mg/kg, *i.v.*) and TH-302 (100 mg/kg, *i.p.*) were then administered 30 min later. **B**, Quantification of the percentage of pimonidazole-positive area from whole tumor sections with (Pyr +) or without (Pyr −) pyruvate injection. Data are mean ± SE of 5 and 3 tumors, respectively. **, *P*<0.01. **C**, Quantification of the percentage of γH2AX-positive area in pimonidazole-positive area with (Pyr +) or without (Pyr −) pyruvate injection. Data are means ± SE of 5 and 3 experiments, respectively. *, *P*<0.05. **D**, Representative images of the caspase activation (green) and the S139 phosphorylation of S139 (pSer^139^) in histone H2AX (red). Part of the nucleus is diminished by potential apoptotic cell death, forming the three-dimensional spaces. **E**, Immunoblotting of phosphorylated histone γH2AX and cleaved caspase-3 from SCCVII tumors 1 day after pyruvate/TH-302 treatment on each day indicated (n = 3). The data are shown as relative intensity to that on day 9. **F**, T_2_ intensity changes 1 day after pyruvate/TH-302 treatment on each day indicated (n = 3–5). Values shown represent means ± SE.

To better understand the mechanisms underlying the potentiation of therapeutic efficacy of TH-302 by pyruvate, immunohistochemical analyses were conducted. As can be seen in **Figure 6A**, the γH2AX stained area, which is elicited by S139 phosphorylation of histone H2AX by DNA damage, was clearly observed within the pimonidazole-positive hypoxic area 24 h after pyruvate/TH-302 administration to SCCVII tumor-bearing mice on day 7. This result indicates that TH-302 is bioreductively activated under relatively more severe hypoxic conditions than pimonidazole. Furthermore, the pimonidazole-positive area in the whole tumor sections increased from 13.8% to 26.5% after pyruvate administration (**Figure 6B**), and the percentage of γH2AX-positive cells within the pimonidazole-positive area increased from 5.8% to 28.2% (**Figure 6C**). To determine the apoptotic effects of TH-302, a FAM-FLIVO™ *in* *vivo* apoptosis assay reagent was injected prior to euthanization to collect tumors 24 h after the combination therapy. Activation of the apoptotic marker caspase-3 was visualized around the DNA-damaged cells, which were identified as the formation of γH2AX-foci, and three-dimensional spaces resulting from decreased cell density by apoptosis, as can be seen on the elimination of DAPI stained nuclei (**Figure 6D**). On immunoblot analysis, increase of both γH2AX and caspase signals were dose- and time-dependent with respect to pyruvate/TH-302 treatment (**Figure S5 in File S1**), suggesting that both DNA damage and subsequent apoptotic cell death are induced by TH-302. As shown in **Figure 6E**, both γH2AX and caspase signals increased one day after treatment of tumors on days 7, 8 and 12, which also correlated with the decrease in the T_2_ intensity on the same days reflecting decreased water content (**Figure 6F**, **Figure S6 in File S1**) [31]. Taken together, these data support the notion that the 2-nitroimidazole trigger in TH-302 is reduced under hypoxia for Br-IPM release and elicit DNA damage and tumor cell death in SCCVII tumor tissues 24 h after combination pyruvate/TH-302, which is consistent with the EPR and MR imaging data.

## Discussion

A *priori* information on the tumor microenvironment, such as the extent of hypoxia or expression levels of radical scavengers like glutathione, is thought to be important for prediction of outcomes following ionizing radiation or HAP treatments. TH-302, a HAP that is selectively activated in hypoxic regions of solid tumors, significantly inhibited the growth of both SCCVII and HT29 cells *in* *vitro* under 0.1% O_2_. Furthermore, the IC_50_ of SCCVII cells was approximately 2.0-fold lower than HT29 cells. This may be because the higher level of glutathione expression in HT29 cells is protective against Br-IPM [21], [32]. By contrast, TH-302 monotherapy showed little growth delay of SCCVII and HT29 tumors *in* *vivo* (**Figure 5**), regardless of its significant cytotoxicity to both cell lines *in* *vitro* (**Figure 1**). This is likely because the median tumor pO_2_ of both tumor xenografts in the 500–1500 mm^3^ size range tested is more than 10 mmHg, with a hypoxic region (<10 mmHg) ratio of less than 50% (**Figure 4** **, Tables S1** and **S2 in File S1**). Under these conditions, the activation level of TH-302 may be inadequate to elicit tumor growth delay *in* *vivo*.

One effective approach to improve the oxygen-dependent therapeutic efficacy of HAP therapy is inhalation of hypoxic gas (e.g. 10% oxygen) [20] but, prolonged exposure to such conditions elicits unwanted side effects, such as transient hypoxemia in live animals. Pyruvate is a major metabolite of glucose in the body. Pyruvate (∼0.43 ml/kg of 230 mM) can be safely administered into patients without significant side effects [33]. In this study, we demonstrated that the exogenous pyruvate can enhance the mitochondrial respiration, resulting in the boost of cellular oxygen consumption in tumor cells (**Figure 2**). Bolus injection of pyruvate into mice with SCCVII or HT29 tumors decreased the median tumor pO_2_ and so increased the hypoxic fraction within 30 min after injection (**Figure 4**). It should be noticed that this effect is transient; tumor pO_2_ returns to the basal level within 5 hours [25]. Three consecutive days of pyruvate/TH-302 combination therapy, started in smaller tumors (∼550 mm^3^) when pyruvate could decrease pO_2_ level to around or less than 10 mmHg, significantly inhibited SCCVII tumor growth (**Figure 5A**), suggesting that the pyruvate-induced transient hypoxia can potentiate the HAP therapy. By contrast, at a later stage of SCCVII tumors with lower pO_2_ values (**Figure 4A**), TH-302 was not significantly affected even though there may be regions hypoxic enough for TH-302 activation (**Figure 4C**). This may be because the larger volume of SCCVII tumors at this stage may impose limits on drug penetration, or there may be necrotic fractions where the nitroimidazole derivatives are unresponsive (**Figure 3**) [30]. On the other hand in HT29 tumors, the median pO_2_ was above 10 mmHg at all tumor sizes measured in this study even after pyruvate injection (**Figure 4B**), explaining no observable benefit on tumor growth delay by the combined pyruvate/TH-302 treatment (**Figure 5B**). These results suggest that non-invasive and quantitative oxygen imaging by EPRI can provide the information of threshold level of the tumor pO_2_ for prediction of the effective therapeutic outcome by HAPs. Furthermore, T_2_ intensity of SCCVII tumors decreased (**Figure 6F**, **Figure S6 in File S1**), correlating with the increase in ADC values as an indicator of edema and cell death (**Figure S7 in File S1**) [34], can be an non-invasive biomarker for tumor response to HAPs, as supported by increased DNA damage and apoptotic cell death (**Figure 6**).

Denko *et al.* reported that dichloroacetate (DCA), an inhibitor of pyruvate dehydrogenase kinase inhibitor 1 (PDK1) that phosphorylates and inhibits pyruvate dehydrogenase from using pyruvate to fuel the mitochondrial TCA cycle, transiently increases cellular oxygen consumption and enhances tumor response to hypoxic cytotoxin PR-104 [35]. Likewise, we have demonstrated that excess pyruvate as a substrate of mitochondrial energy metabolism can enhance oxygen consumption, increasing transient tumor hypoxia for TH-302 sensitization. Taken together, it is clear that pharmacologically targeted mitochondrial pyruvate flux can improve antitumor efficacy of HAPs by increasing transient tumor hypoxia.

Collectively, the EPRI and MRI methods provided useful information pertaining to tumor pO_2_ status and longitudinal drug response. The EPRI studies revealed hypoxia-dependent activation of TH-302, which can be potentiated using approaches to transiently increase hypoxia. It should be noted that the pyruvate-induced hypoxia in solid tumors may provide benefit if combined with any bioreductive prodrug including DNA-reactive cytotoxins or other hypoxia-activated functional molecules.

## Supporting Information

File S1 **This file contains Figures S1–S7 and Tables S1–S3.** Figure S1. A scheme of the TH-302 (A) or pimonidazole (B) reductive activation pathway. One-electron reduction of nitroimidazole in each compound produces a radical anion intermediate, which can undergo futile redox cycling under normoxic conditions to generate superoxide, or fragmentation or further reduction under hypoxic conditions. Figure S2. Cell viability following a 2 h treatment of SCCVII and HT29 cells with varying concentrations of pyruvate under aerobic (21% oxygen) conditions. A, B, Cell viability of SCCVII (A) or HT29 (B) cells following a 2 h treatment with varying concentrations of pyruvate at 21% O_2_. Data are from 6 replicates; error bars represent the SE. Figure S3. Three-dimensional oxygen image in a SCCVII tumor using EPRI and MRI before and after pyruvate/TH-302 injection. A, T_2_-weighted anatomical image and pO_2_ maps measured before and 30 min after pyruvate injection in a representative SCCVII tumor-bearing mouse 7 days after tumor implantation. The T2 map was obtained before and 1 day after treatment with TH-302. B, T_2_-weighted anatomical image and pO_2_ maps measured before and after three consecutive days (days 7, 8 and 9) of TH-302 monotherapy. T2 map was obtained before and after three times TH-302 treatment. Figure S4. Percentage body weight change of model mice in each treatment group. A, C3H/Hen mice bearing murine SCCVII tumors (n = 5). B, Athymic NCr-nu/nu nude mice bearing human HT29 tumors. Figure S5. Immunoblotting of histone H2AX and caspase-3. A, Immunoblotting of phosphorylated S139 (pSer^139^) in histone H2AX and cleaved caspase-3 from SCCVII tumors at indicated times (h) after pyruvate/TH-302 treatment on day 7 (n = 3). B, Time-dependent increases in phosphorylation of H2AX (red) and cleavage of caspase-3 (green). C, TH-302 dose-dependent increases in phosphorylation of H2AX (red) and cleavage of caspase-3 (green). Figure S6. A, B, T_2_-weighted anatomical images and T2 maps scanned before (A) and 1 day after (B) pyruvate/TH-302 treatment in a representative SCCVII tumor bearing mouse on the indicated days after tumor implantation. Figure S7. Noninvasive detection of treatment response by MRI. A–B, T_2_-weighted anatomical, ADC and T2 maps measured before (A) and 1 day after (B) pyruvate/TH-302 treatment on a representative SCCVII tumor-bearing mouse 7 days after tumor implantation. C, Relative ADC changes with (Test) or without (Cont) pyruvate/TH-302 treatment. ΔADC (mm^−3^/s)  =  ADC (day8) – ADC (day7). Data are means ± SE of 4 experiments. *, *P*<0.05. D, Relative T2 intensity changes with (Test) or without (Cont) pyruvate/TH-302 treatment. ΔT2 (ms)  =  T2 (day8) – T2 (day7). Data are means ± SE of 4 experiments. **, *P*<0.01. Table S1. Median pO_2_ value before (0 min) and 30 min after pyruvate treatment on SCCVII and HT29 tumors. Table S2. Percentage of hypoxic fraction (HF) of <15.2 mmHg or <10 mmHg before (0 min) and 30 min after pyruvate treatment on SCCVII and HT29 tumors. Table S3. Microsatellite allele marker profile of the SCCVII cell line.
(DOCX)
