# Supplementary material for: Pyruvate Induces Transient Tumor Hypoxia by Enhancing Mitochondrial Oxygen Consumption and Potentiates the Anti-Tumor Effect of a Hypoxia-Activated Prodrug TH-302
Source: PLoS One. 2014 Sep 25;9(9):e107995. doi: 10.1371/journal.pone.0107995 (PMC4177858; doi:10.1371/journal.pone.0107995)
Supplement: File S1 — This file contains Figures S1–S7 and Tables S1–S3. Figure S1. A scheme of the TH-302 (A) or pimonidazole (B) reductive activation pathway. One-electron reduction of nitroimidazole in each compound produces a radical anion intermediate, which can undergo futile redox cycling under normoxic conditions to generate superoxide, or fragmentation or further reduction under hypoxic conditions. Figure S2. Cell viability following a 2 h treatment of SCCVII and HT29 cells with varying concentrations of pyruvate under aerobic (21% oxygen) conditions. A, B, Cell viability of SCCVII (A) or HT29 (B) cells following a 2 h treatment with varying concentrations of pyruvate at 21% O2. Data are from 6 replicates; error bars represent the SE. Figure S3. Three-dimensional oxygen image in a SCCVII tumor using EPRI and MRI before and after pyruvate/TH-302 injection. A, T2-weighted anatomical image and pO2 maps measured before and 30 min after pyruvate injection in a representative SCCVII tumor-bearing mouse 7 days after tumor implantation. The T2 map was obtained before and 1 day after treatment with TH-302. B, T2-weighted anatomical image and pO2 maps measured before and after three consecutive days (days 7, 8 and 9) of TH-302 monotherapy. T2 map was obtained before and after three times TH-302 treatment. Figure S4. Percentage body weight change of model mice in each treatment group. A, C3H/Hen mice bearing murine SCCVII tumors (n = 5). B, Athymic NCr-nu/nu nude mice bearing human HT29 tumors. Figure S5. Immunoblotting of histone H2AX and caspase-3. A, Immunoblotting of phosphorylated S139 (pSer139) in histone H2AX and cleaved caspase-3 from SCCVII tumors at indicated times (h) after pyruvate/TH-302 treatment on day 7 (n = 3). B, Time-dependent increases in phosphorylation of H2AX (red) and cleavage of caspase-3 (green). C, TH-302 dose-dependent increases in phosphorylation of H2AX (red) and cleavage of caspase-3 (green). Figure S6. A, B, T2-weighted anatomical images and T2 maps scanned [file pone.0107995.s001.docx]

**Supporting Information (Takakusagi et al. for PLoS One)**

**Figure S1**.

**A scheme of TH-302 (A) or pimonidazole (B) reductive activation pathway.** One-electron reduction of nitroimidazole in each compound produces a radical anion intermediate, which can undergo futile redox cycling under normoxic conditions to generate superoxide, or fragmentation or further reduction under hypoxic conditions.


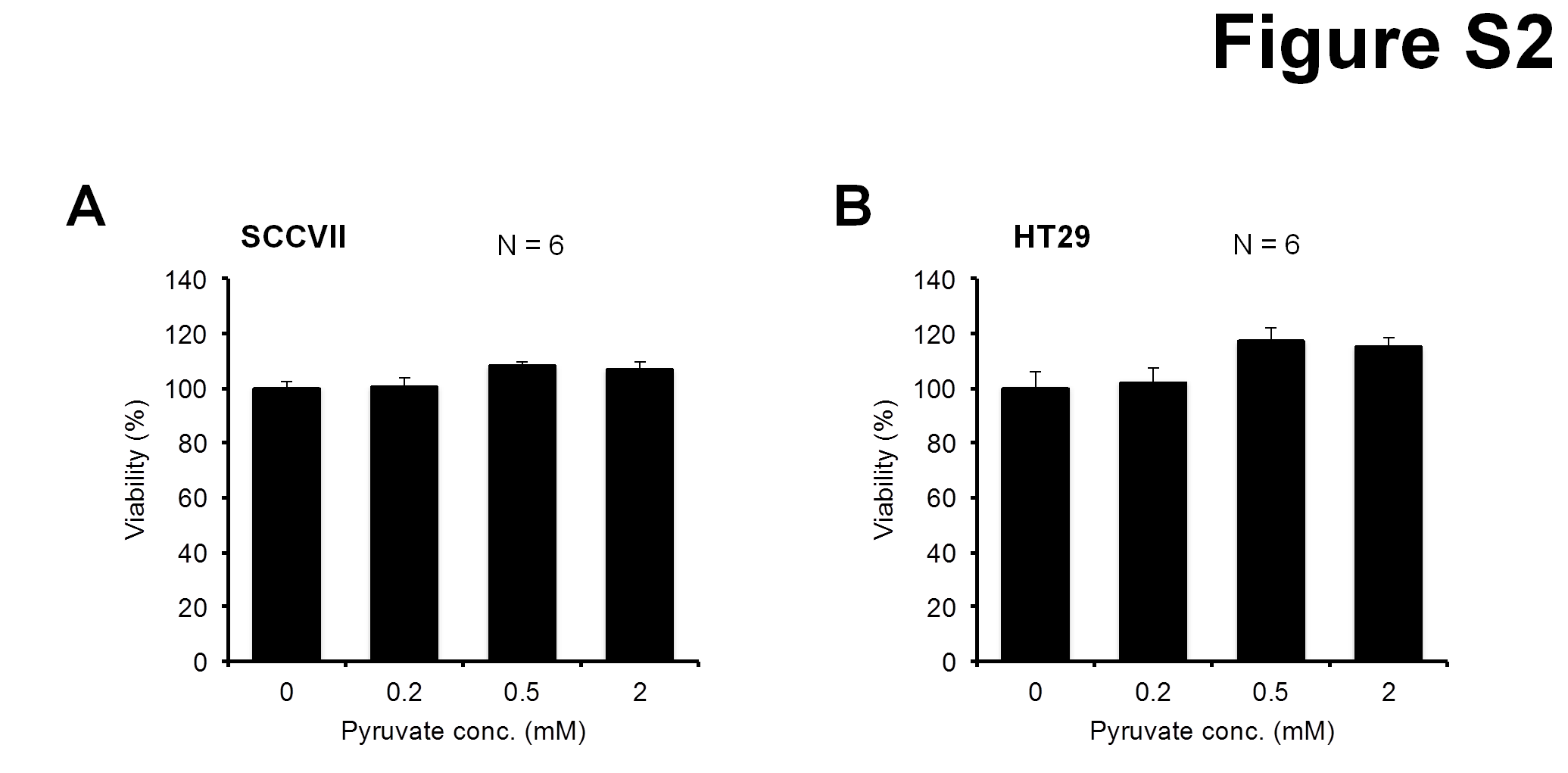


**Figure S2.**

**Cell viability following a 2 h treatment of SCCVII and HT29 cells with varying concentrations of pyruvate under aerobic (21% oxygen) conditions.** **A, B,** Cell viability of SCCVII (**A**) or HT29 (**B**) cells following a 2 h treatment with varying concentrations of pyruvate at 21% O_2_. Data are from 6 replicates; error bars represent the SE.


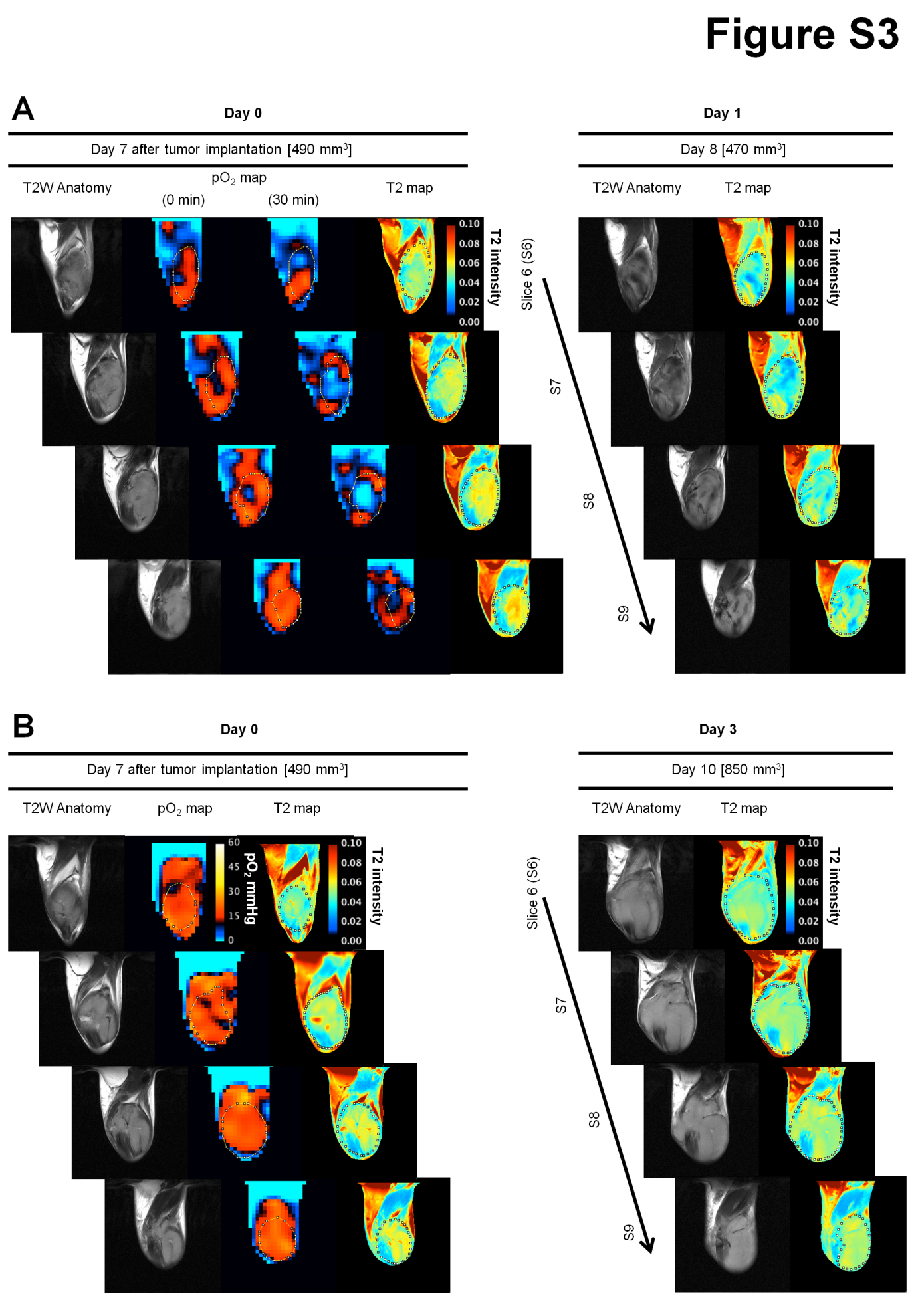


**Figure S3**.

**Three-dimensional oxygen image in a SCCVII tumor using EPRI and MRI before and after pyruvate/TH-302 injection.** **A**, T_2_-weighted anatomical image and pO_2_ maps measured before and 30 min after pyruvate injection in a representative SCCVII tumor-bearing mouse 7 days after tumor implantation. The T_2_ map was obtained before and 1 day after treatment with TH-302. **B**, T_2_-weighted anatomical image and pO_2_ maps measured before and after three consecutive days (days 7, 8 and 9) of TH-302 monotherapy. T_2_ map was obtained before and after three times TH-302 treatment.


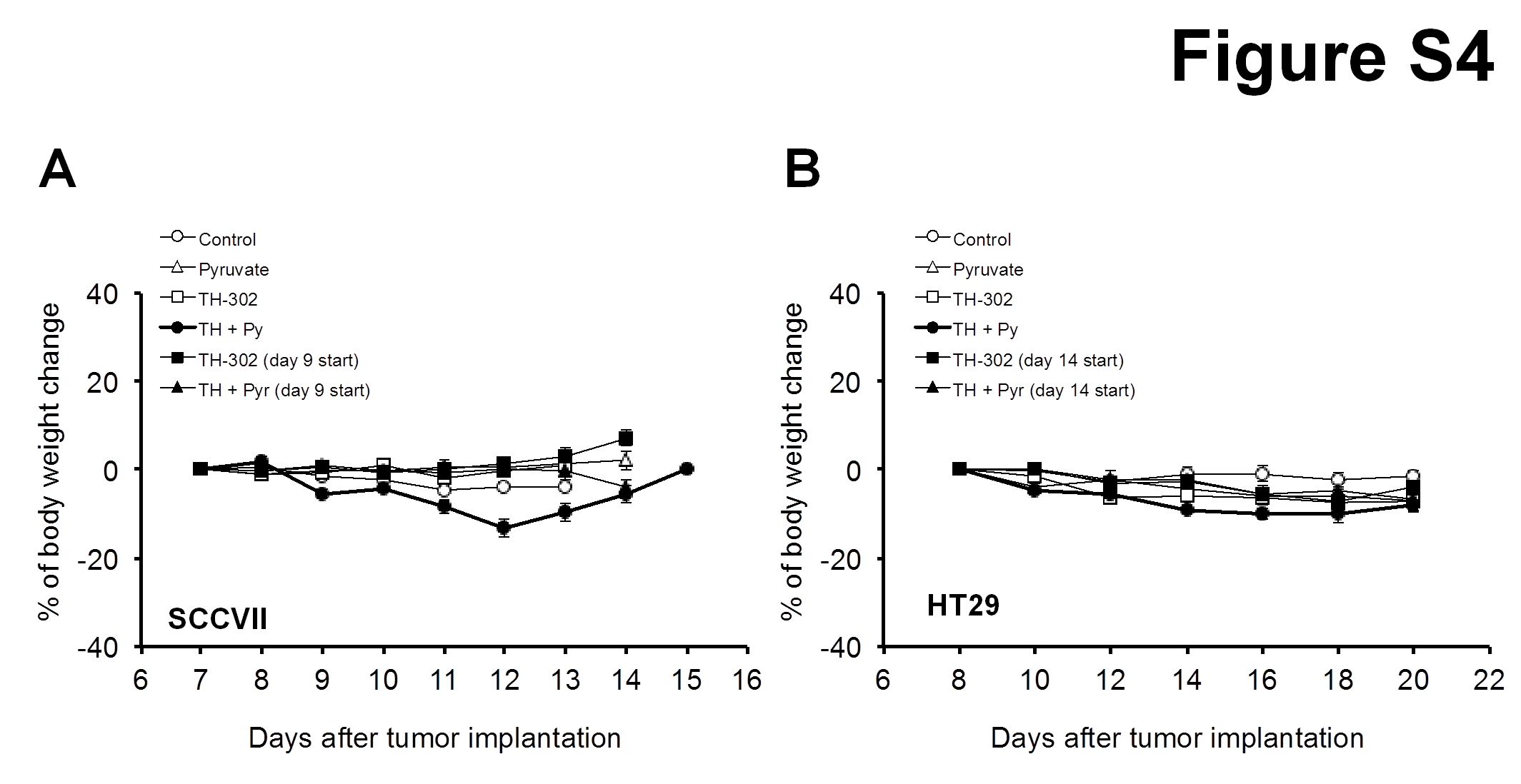


**Figure S4.**

**Percentage body weight change of model mice in each treatment group. A**, C3H/Hen mice bearing murine SCCVII tumors (n = 5). **B**, Athymic NCr-nu/nu nude mice bearing human HT29 tumors.


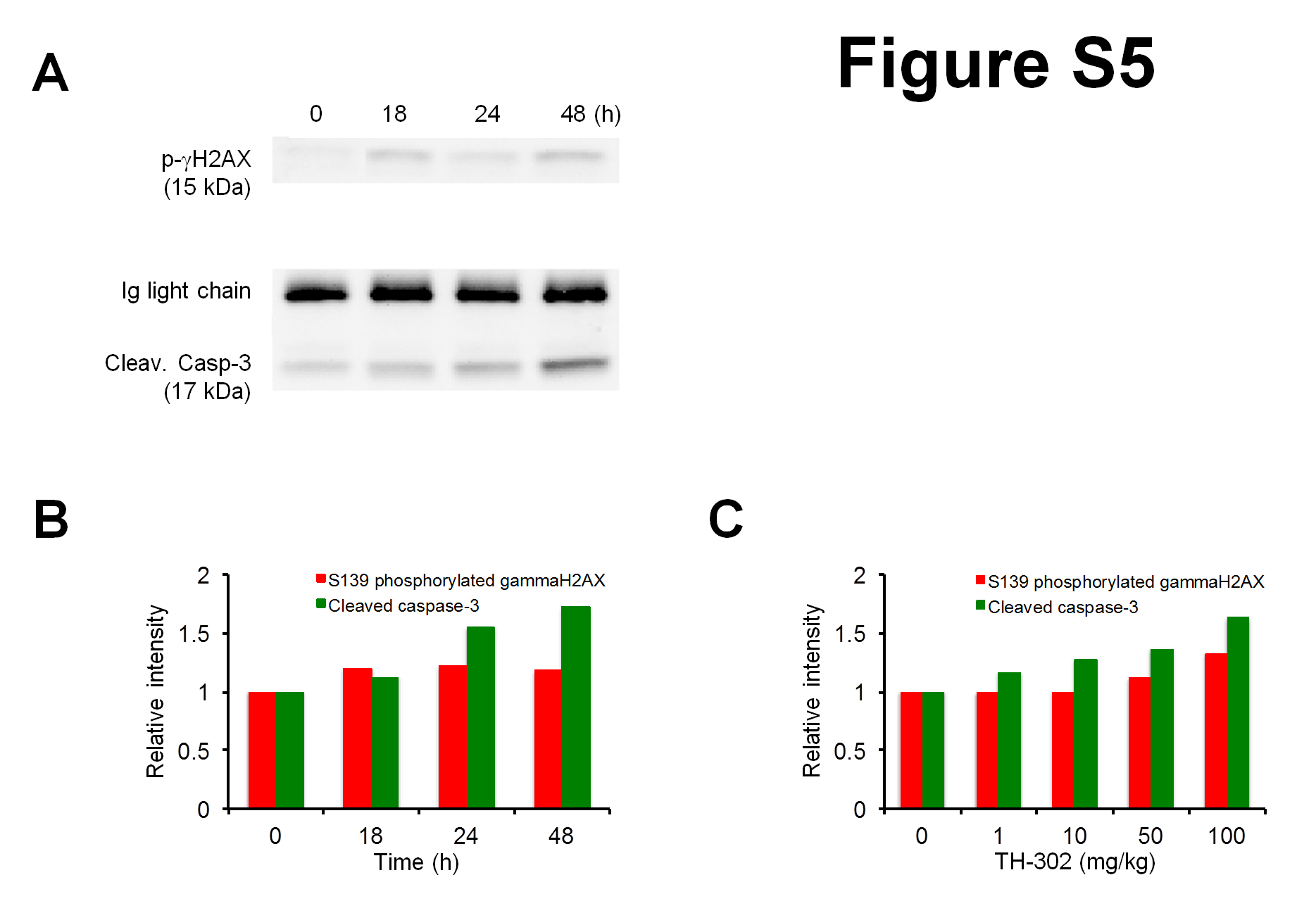


**Figure S5.**

**Immunoblotting of histone H2AX and caspase-3. A**, Immunoblotting of phosphorylated S139 (pSer^139^) in histone H2AX and cleaved caspase-3 from SCCVII tumors at indicated times (h) after pyruvate/TH-302 treatment on day 7 (n = 3). **B**, Time-dependent increases in phosphorylation of H2AX (red) and cleavage of caspase-3 (green). **C**, TH-302 dose-dependent increases in phosphorylation of H2AX (red) and cleavage of caspase-3 (green).


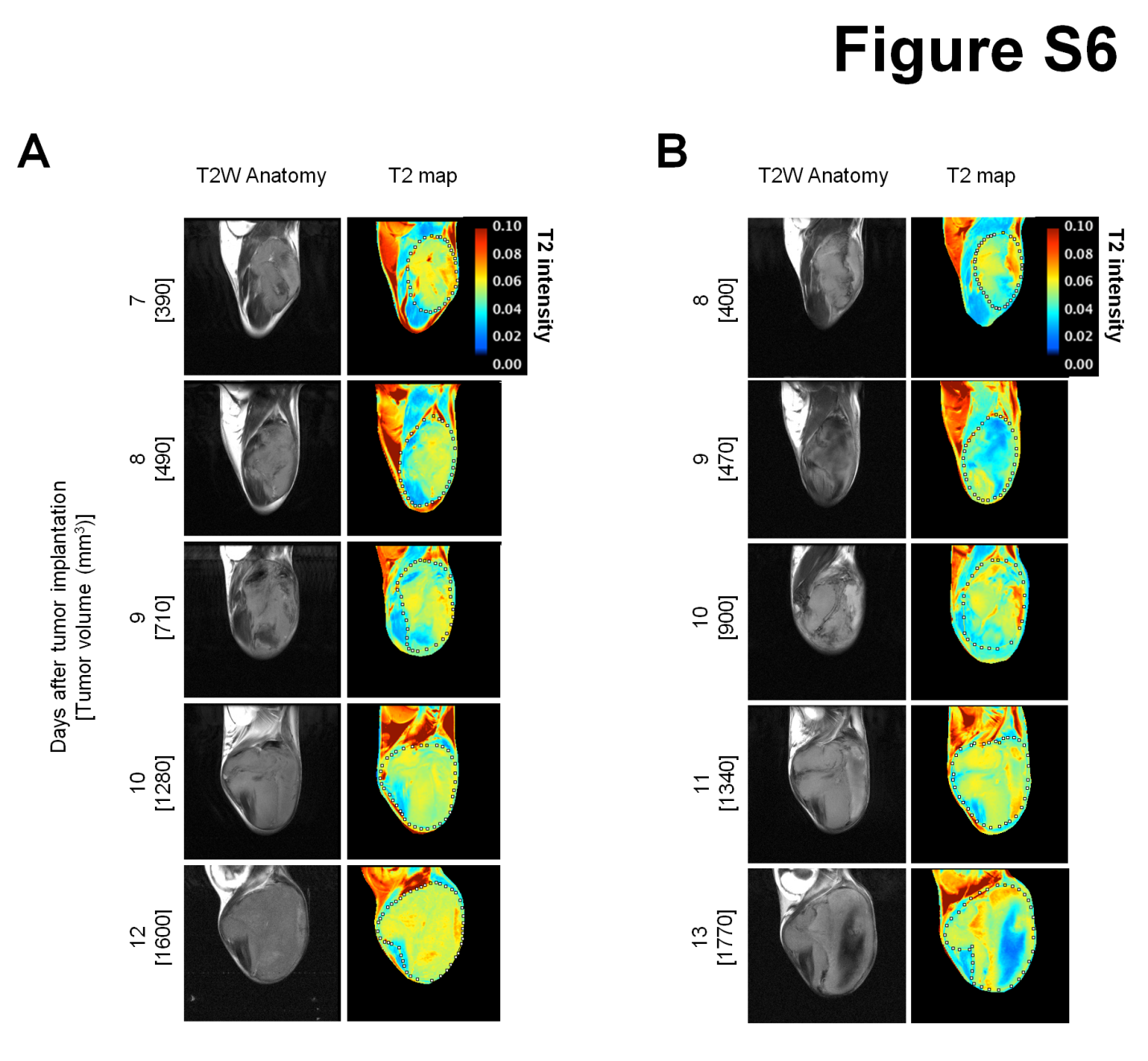


**Figure S6**.

**A, B, T_2_-weighted anatomical images and T_2_ maps scanned before (A) and 1 day after (B) pyruvate/TH-302 treatment in a representative SCCVII tumor bearing mouse on the indicated days after tumor implantation.**


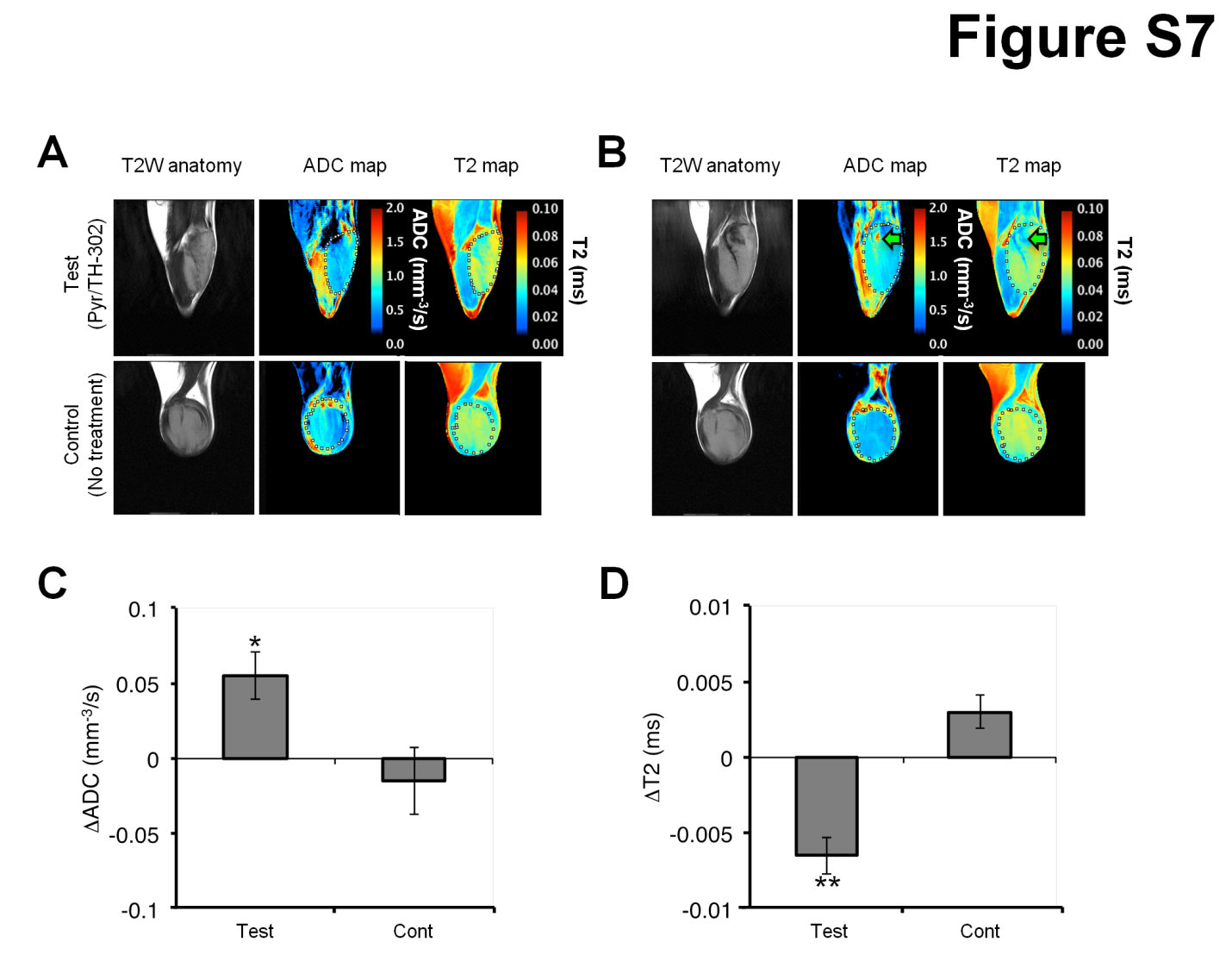


**Figure S7.**

**Noninvasive detection of treatment response by MRI.** **A-B**, T_2_-weighted anatomical, ADC and T2 maps measured before (**A**) and 1 day after (**B**) pyruvate/TH-302 treatment on a representative SCCVII tumor-bearing mouse 7 days after tumor implantation. **C**, Relative ADC changes with (Test) or without (Cont) pyruvate/TH-302 treatment. ΔADC (mm^-3^/s) = ADC (day8) – ADC (day7). Data are means ± SE of 4 experiments. *, *P* <0.05. **D**, Relative T_2_ intensity changes with (Test) or without (Cont) pyruvate/TH-302 treatment. ΔT_2_ (ms) = T_2_ (day8) – T_2_ (day7). Data are means ± SE of 4 experiments. **, *P* <0.01.


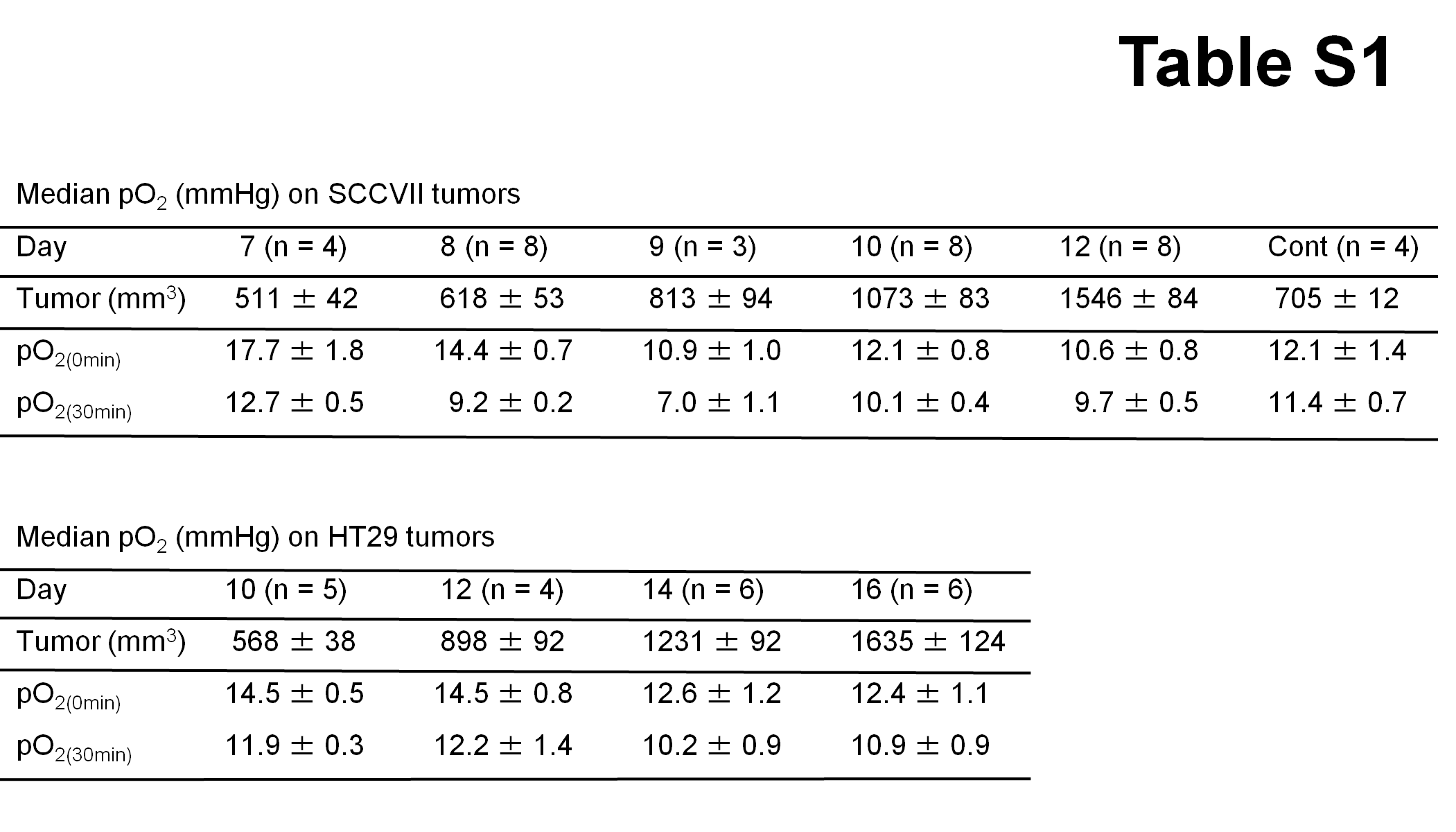


**Table S1.**

**Median pO_2_ value before (0 min) and 30 min after pyruvate treatment on SCCVII and HT29 tumors.**


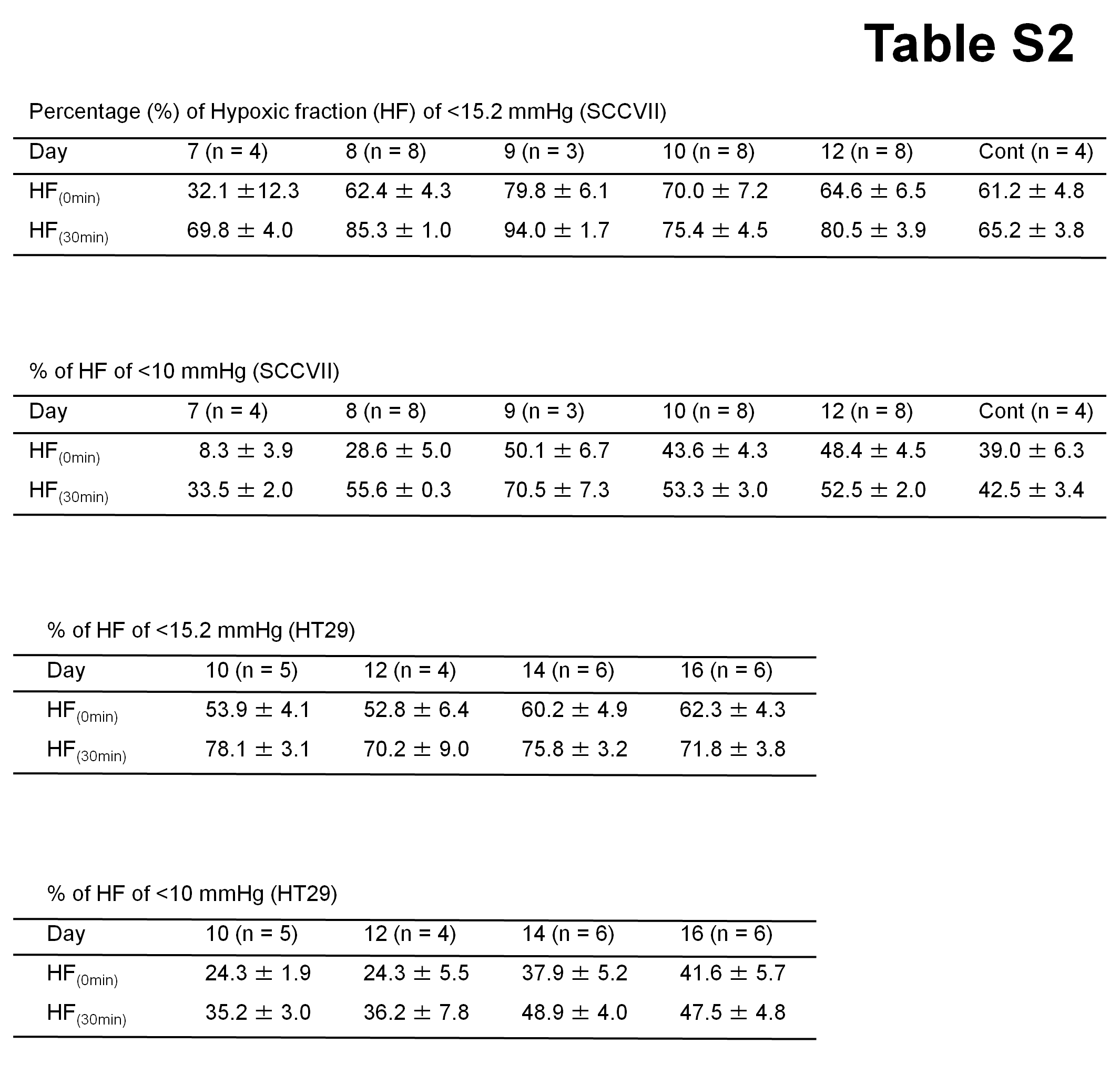


**Table S2.**

**Percentage of hypoxic fraction (HF) of <15.2 mmHg or <10 mmHg before (0 min) and 30 min after pyruvate treatment on SCCVII and HT29 tumors.**


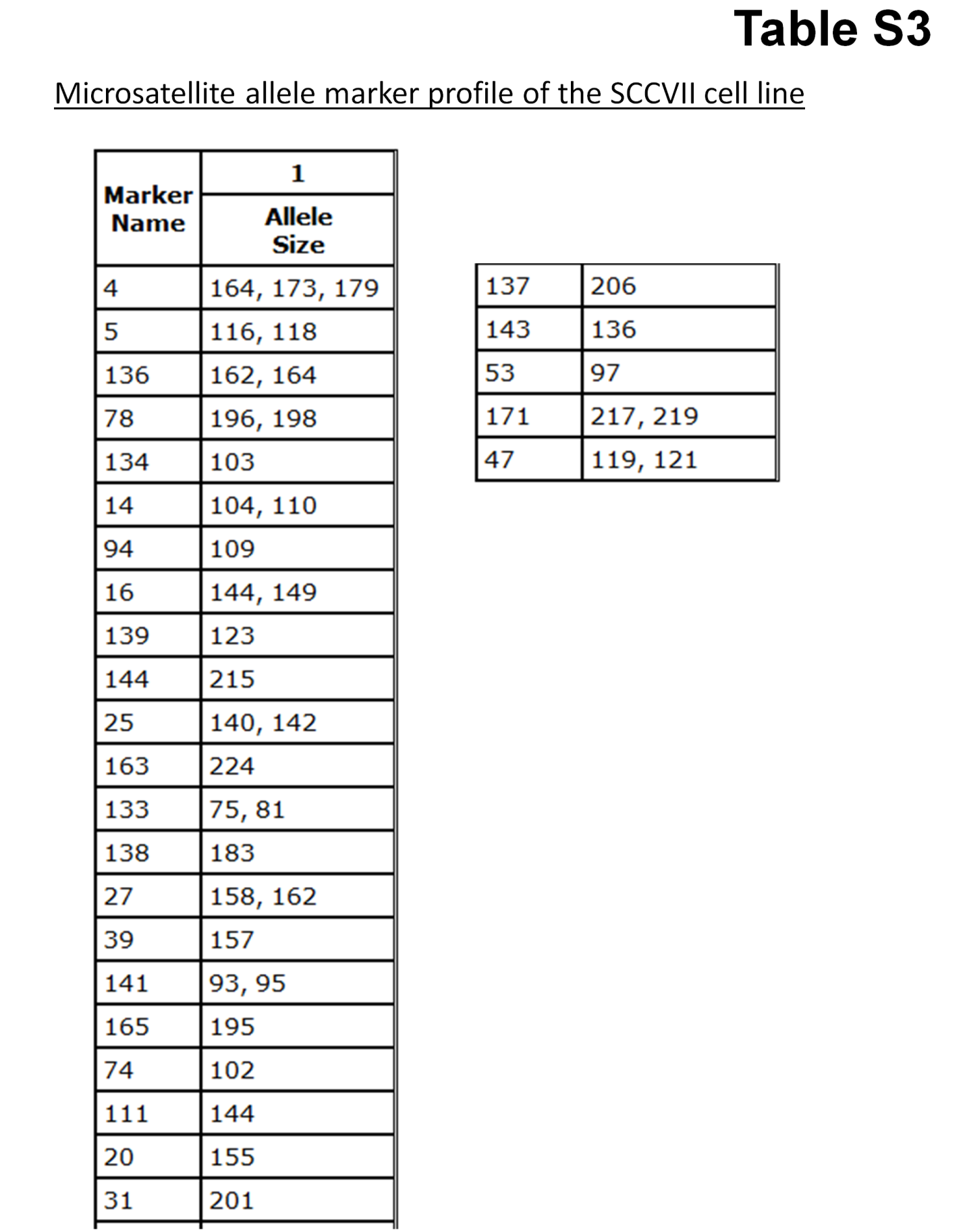


**Table S3.**

**Microsatellite allele marker profile of the SCCVII cell line**
